# The working road map in a neurosurgical Hybrid Angio-Surgical suite------ development and practice of a neurosurgical Hybrid Angio-Surgical suite

**DOI:** 10.1186/s41016-017-0108-1

**Published:** 2018-03-22

**Authors:** Zeguang Ren, Shuo Wang, Kaya Xu, Maxim Mokin, Yuanli Zhao, Yong Cao, Jia Wang, Hancheng Qiu, Siviero Agazzi, Harry van Loveren, Jizong Zhao

**Affiliations:** 1 Department of Neurosurgery, University of South Florida, 2 Tampa General Circle, 7th floor, Tampa, FL 33606 USA; 2 Department of Neurosurgery, Beijing Tiantan Hospital, Beijing, 100050 China; 3 China National Clinical Research Center for Neurological Diseases, Beijing Tiantan Hospital, Beijing, China; 4 Department of Neurosurgery, Guiyang Medical University, Guiyang, 550004 China

**Keywords:** Hybrid, Angiogram, Operating room, Cerebrovascular

## Abstract

**Background:**

The concept of a Hybrid Angio-Surgical Suite (HASS) has emerged as a solution to the complexity of cerebrovascular surgery and the need for immediate intraoperative feedback. When to use it, what cases are suitable for its use, who can use it and how to use it remain debatable.

**Objective:**

Provide the information regarding the application of the HASS for hospital, neurosurgeon and interventionalist.

**Methods:**

We review the literatures of case reports and studies on the use of the hybrid angio-sugical suite along with application of HASS in our own practice.

**Results:**

Indications for using HASS on different types of cerebral vascular disease, including cerebral aneurysm, AVM, DAVF, carotid and vertebral stenosis/occlusion, are addressed. The application of HASS for other non-cerebral vascular diseases, such as trauma, spine and skullbase cases, is reviewed and discussed.

**Conclusion:**

HASS has made many surgical procedures safer and many difficult or previously untreatable conditions much more tractable and cost-effective. Other than used in cerebral vascular disease, HASS has much more applications, such as trauma, spine and other neurosurgical diseases.

## Background

The concept of a Hybrid Angio-Surgical Suite (HASS) emerged as a solution to the complexity of cerebrovascular surgery and the need for immediate intraoperative feedback. It combines the capacity of both operating room and interventional suite --- a standard operating room equipped with a biplane or single plane angiogram machine. Early studies with intraoperative portable digital subtraction angiography (DSA) — early “hybrid surgery” for different cerebrovascular diseases -- showed that the intraoperative angiogram (IOA) altered surgical management, frequently by avoiding additional surgery [1–5]. With the recent enormous advancement of angiographic equipment, and hybrid operating tables, endovascular products and techniques, the combined multi-modality treatment with hybrid techniques has significantly improved patient outcomes and made many previously untreatable conditions treatable [5–8]. The hybrid angio/surgery suite is becoming a standard facility for many hospitals around the world. Many neurosurgeons and neurointerventionists use it for treatment of cerebrovascular diseases, but when to use it, what cases are suitable for its use, who can use it and how to use it remain debatable. In this paper, we examine the literature on case reports and series using the hybrid angio-surgical suite. We discuss the indications for using HASS on different types of cerebral vascular disease including cerebral aneurysm, arteriovenous malformation (AVM), dural arteriovenous fistula (DAVF), carotid stenosis/occlusion and other non-cerebral vascular diseases.

## Application of HASS in aneurysm treatment

The routine or selective use of the IOA in aneurysm surgery has long been advocated because of its low risk-to-benefit ratio [1–4, 9–12]. The 3D rotational angiography and flat-panel detector computed tomography have further advanced this intraoperative armamentarium since the 1960s by its rapid image acquisition and ability to overcome the artifacts from aneurysm clips and coils [9–12]. Chiang et al. [2] reported repositioning of the clip in 11% of the aneurysms clipped because of residual aneurysms (6.5%), parent vessel occlusion (3%) or both (1.5%), after discovery with routine use of IOA. Tang et al. [3] reported positive IOA findings in 12.4% of aneurysms clipped, which prompted changes in their surgical strategies.

Currently, HASS was commonly used for hybrid treatment of giant brain aneurysms, especially giant paraclinoid and basilar tip aneurysms. These aneurysms tend to rupture intraoperatively, as safe dissection is often limited by the mass effect of the aneurysms. In these cases, flow arrest and decompression of aneurysms are achieved in several ways, such as occlusion of common carotid artery (CCA) and external carotid artery (ECA) for retrograde suction [13], proximal balloon occlusion [14, 15]. In these early reports, the authors placed the balloon into the proximal parent artery, whereas in those of Thorell et al. [16] and Steiger et al. [17], the occlusion was performed intracranially below the aneurysmal orifice or neck. Hu et al. [18] first reported a combination of endovascular and surgical approaches to treat giant ophthalmic artery aneurysms in a hybrid operating theater. They used a dye in the contrast to visualize the balloon, allowing for improved accuracy in placement of the balloon at the orifice of the aneurysm. This technique virtually removes the risk of intraoperative rupture because there is no blood flow into the aneurysm after the balloon is fully inflated.

Another advantage of HASS in the treatment of aneurysms is the immediate resue of complications from intitial procedures (either of endovascular embolization or surgical clipping). Qiu et al. [15] reported immediate rescue craniotomy for clipping following intraoperative rupture of middle cerebral artery (MCA) aneurysm embolization; Elhammady et al. [6] reported a salvage procedure in which an adjuvant endovascular balloon was used for proximal and distal control for an unexpected and uncontrolled rupture during clipping of a 2 cm paraclinoid aneurysm. Although this wasn’t done in a HASS, immediate availability of endovascular support can be used in similar situatiosn with improved outcomes.

## Application of HASS in AVM treatment

Currently there is no consensus on AVM treatment, especially for the Spetzler-Martin (SM) grade III to V patients. Embolization prior to surgery is typically done in patients with high SM grade lesions in an effort to decrease the size of the malformation and to eliminate high-risk characteristics, including aneurysms and fistulas [19]. A decrease in complications and improvement in outcomes can be expected by eliminating difficult surgical areas and reducing the intraoperative bleeding and operation times with pre-op embolization. Normally pre-op embolization for an un-ruptured AVM is done prior to surgery in separate single or multiple sessions. Thus, HASS is not generally used for the pre-op embolization. But when intro-procedural rupture happens, HASS will make a difference for the patient’s outcome. Dyna CT of the angiogram machine is available to detect a hematoma. CT guided, external ventricular drainage placement can be performed instantly, and subsequent craniotomy can be done immediately.

The most useful function of HASS for AVM surgery is to confirm complete resection of an AVM nidus to avoid repeat surgery. Many studies have shown the necessity of IOA to avoid repeated surgery for residual AVM [1, 19–21]. In Kotowski’s series [22], in 16% of cases (22% for unruptured AVM and 13% for ruptured AVM), intraoperative imaging visualized AVM remnants ≤3 mm and allowed for completion of the resections in the same sessions which was missed on Indocyanine Green (ICG) angiography. The authors concluded that ICG angiography is recommended as a complement rather than as a replacement of DSA. It is believed that difficulties in visualization of remnants in ICG angiography are generally due to the small size, not to a deeper location. Modern HASS with flat panel detector coupled to a frameless neuronavigation system can guide the localization of the vascular lesion, such as residual AVM with intraoperatively acquired Dyna CT/XperCT and 3D rotational angiography datasets. Neuronavigation can be updated at any time by co-registration with intraoperative 3D rotational angiography or Dyna CT/XperCT datasets.

One specific major advantage of HASS is in the management of vascular emergency interventions. For patients suffering from a ruptured AVM with intracerebral hemorrhage and mass effect with herniation, the hybrid OR will make a big difference in outcome. In these cases, hematoma evacuation needs to be done urgently, but a high quality angiogram is also needed for assessing the AVM nidus for further surgical intervention after successful hematoma evacuation. The dedicated HASS setup has made it possible for the surgeon to assess the hematoma by Dyna CT and AVM angiostructure by intraoperative 3D angiogram, continue emergent treatment by microsurgical removal of the intracerebral hematomaand and the AVM, and for final confirmation of complete resection – all without the necessity of additional transport of the patient.

## Application of HASS for DAVF treatment

Currently, with advanced endovascular techniques and embolic material such as Onyx, the majority of DAVFs can be treated successfully by endovascular embolization [23], but surgical obliteration is still important and necessary when the endovascular approach fails due to anatomic features. Frequently, for complex and deep seated DAVFs it is difficult to ensure surgical access without very invasive cranial base approaches [24]. Regular transarterial and transvenous approaches also may fail due to occlusion of the sinus and tortuosity of arterial feeders. In this situation, a hybrid procedure with open surgery to provide alternative access for endovascular embolization would be the only option. Surgical exposure of vessels leading to (feeders) or from the fistula (draining vein) allow more effective embolization [25]. Hybrid surgery combining a different craniotomy and endovascular embolization provides a less invasive and more feasible alternative for the treatment of difficult DAVFs. In general, hybrid surgery provides access for intraoperative embolization with the following approaches:

1. Cortical vein access: the dilated cortical vein can be accessed through a small craniotomy without the need for accessing the whole DAVF, making the approach much less invasive. as reported by Shen et al. [26]. In their case the periclival venous plexus was approached intradurally through a keyhole pterional craniotomy, direct puncture of the venous pocket was performed with 18 gauge spinal needle, and the fistula was embolized completely with NBCA. Minimally invasive surgical procedures, such as a stereotactically placed burr hole, can allow direct puncture of a cortical draining vein or the fistula itself [7, 8].

2. Arterial feeder access: oftentimes the proximal part of fistula feeders such as the meningeal artery or occipital artery are extremely tortuous. Access to the distal end of feeders would be impossible without a surgical approach. In Lin’s reports [27], they treated a Borden III DAVF in HASS after conventional trans-venous and trans-arterial routes failed. The major middle meningeal artery feeder was accessed directly through a temporal craniotomy.

3. Venous sinus access: transverse-sigmoid sinus DAVF frequently has sigmoid or transverse sinus occlusion. The contralateral transverse sinus is not always available for access, and multiple small arterial feeders make the hybrid treatment the only option. Transcranial exposure of large dural venous sinuses for surgically-assisted direct transvenous embolization of high-grade dural arteriovenous fistulas has been reported [28, 29] including our case. This is often the last resort for the treatemtn of high grade complicated DAVF.

4. Ophthalmic vein access: access to carotid-cavernous fistula (CCF) for embolization is frequently limited. CCF can be treated transarterially and transvenously. However, when both approaches had failed, a percutaneous trans-orbital approach has been used by us and many providers [30–32]. It is recommended to do it with an ophthalmologist in case the patient develops a retro-orbital hematoma. Direct cut-down to the superior ophthalmic vein via a small incision on the eyelid or facial vein can be performed for accessing CCF with its successful embolization [33–36].

## Application of HASS for carotid artery diseases

For carotid artery disease, the endarterectomy (CEA) is normally the first treatment option, with carotid stenting being an alternative option for cases with contraindications for CEA. In routine CEA, IOA isn’t regularly used; however, in some difficult cases, the HASS may be the only option, such as cases with long segment stenosis extending distally to the skull base and proximally to the origin site of CCA.

László Pintér [37] first reported a hybrid procedure for a long, total occlusion of the CCA that combines the use of internal carotid artery (ICA) CEA and the use of retrograde ring-stripping of the CCA and angioplasty/stenting of residual stenosis at the origin of the vessel. The hybrid procedure offers the surgeon control against distal emboli and angiographic guidance during the retrograde thromboendarterectomy. Shin et al. [38] used direct exposure of the common carotid artery for carotid angioplasty/stenting due to tortuous vasculature access via the femoral route. Simultaneous CEA and CAS for high-grade bilateral carotid stenosis has been reported by Xu et al. [39]

Symptomatic chronic total occlusion of the carotid artery presents another challenging situation. A few cases of endovascular angioplasty for total occlusion of ICA have been reported [40, 41], but it is a challenging procedure. Jiao’s group described a large series of recanalization of chronic carotid artery occlusions with carotid endarterectomy or hybrid procedure [42], which include Fogarty catheter embolectomy and carotid angioplasty/stenting. The hybrid procedure makes recanalization of long segment occlusions possible.

As indicated in the Shih’s report [43], hybrid surgery has a lot of advantages in comparison to the femoral endovascular approach. The hybrid technique allows insertion of the endovascular sheath into the true lumen with direct visualization, possibly saving operating time that would otherwise be spent on attempting blind recanalization in the occluded ICA segment. Another advantage is the avoidance of distal embolic stroke due to occlusion of the common carotid artery.

## Application of HASS to other non-vascular diseases

The HASS is equipped with a single or biplane high-resolution fluoroscopy with flat panel detectors. It can function as a “portable fluoroscope” in the OR for different purposes. In general, X-rays were used in the OR for identifying different anatomic targets for localization. Compared to regular portable fluoroscopy, HASS has several advantages: being integrated into the OR setting and workflow, it is available whenever needed, eliminating the wait for a technician; surgeons can operate the angio machine by themselves in a sterile fashion, improving efficiency and accuracy; the image quality of the angiosuite machine is much better than that of the OR portable fluoroscope, which is critical for target localization; the soft tissue/bone contrast is superior, and the details of the target can be seen clearly, such as the foramen ovale and a pedicle of the spine. With its clear advantages, many procedures will benefit from being performed in HASS.

1. Penetrating head and neck trauma: Penetrating head trauma is usually deep and close to or directly invading the intracranial/extracranial vessels. The availability of an occlusion balloon in HASS for penetrating object removal makes the surgery safer in case of vessel injury. Endovascular treatment for unexpected vessel injury after object removal may be the only option for the patient to survive. Nguyen et al. [44] reported a case of a ballpoint pen penetrating the left nostril and going through the right superior orbital fissure and cavernous sinus, and injuring ICA in HASS.

2. Trigeminal rhizotomy: Percutaneous trigeminal rhizotomy (PTR) remains one of the most common surgical treatments for trigeminal neuralgia. Rose et al. were the first to report the use of high resolution biplane neuroangiosuites in PTR [45]. This technique has many advantages over c-arm fluoroscopy for PTRs such as fewer cannulation attempts and potentially shorter operative times, making the procedure faster and easier in HASS.

3. Spine surgery almost always uses intraoperative X-ray for localization. Accurate localization of bony structures is critical during spine surgery to avoid complications. Ease of operation of the angio fluoroscope will save significant operative time, decrease radiation dose to the patient and more importantly improve the accuracy of localization of the bony target especially for the screw placement. Richter et al. reported that after 1 year of using the hybrid OR in orthopedic trauma, they saw no increase of complication rates and were more confident when placing spine implants in both minimally invasive and conventional procedures [46]. As more spine surgeons learn of the advantages of HASS, we expect that the application of hybrid OR for spine surgery would become a standard setting.

4. Skull base lesions are often close to major arterial and venous structures, increasing the chance of iatrogenic injury to these large vessels. During skullbase procedures like transphenoidal/transnasal approach for intraseller and suprasellar lesions there is a possibility of intraoperative cavernous ICA injury or basilar artery injury, which is associated with high mortality/morbidity, mandating immediate hemostasis and/or injury repair. Endovascular deployment of covered stents or balloon-assisted primary repair for intragenic ICA injury is most often performed [47] and has a high success rate.

## Conclusion

HASS has many proven and potential advantages for treating different cerebrovascular and non-cerebrovascular diseases (see Table 1).

**Table 1:** Suggested indications for using hybrid angio-surgical suite

Medical conditions	Indications
Aneurysm	• Post-clipping review for residual aneurysm and patency of parent vessels [2, 3] • Balloon temporal occlusion for proximal control [13–18] • Immediate rescue/salvage procedures [6, 15]
AVM	• Post-resection checking for residual AVM [1, 19–21] • Investigating ruptured AVM with herniation • Emergent management of intraoperative rupture of AVM embolization • Acquired IOA for navigation of residual AVM
Carotid and vertebral artery disease	• Provide direct access to CCA/ICA/VA for further endovascular intervention [37–39] • Symptomatic total occlusion of CCA/ICA/VA with distal long segment occlusion/stenosis [40–42]
DAVF	• Provide alternative access for endovascular embolization, including cortical vein, arterial feeders, venous sinus and ophthalmic vein [7, 8, 25–36]
Other non-cerebrovascular diseases	• Penetrating trauma to head and neck [44] • Spinal fusion [46] • Percutaneous trigeminal rhizotomy [45] • Skull base surgery [47]

## Abbreviations

AVM: Arteriovenous malformation
CCA: Common carotid artery
CCF: Carotid-cavernous fistula
CEA: Carotid Endarterectomy
DAVF: Dural arteriovenous fistula
DSA: Digital subtraction angiography
ECA: External carotid artery
HASS: Hybrid angio-surgical suite
ICA: Internal carotid artery
IOA: Intraoperative angiogram
MCA: Middle cerebral artery
PTR: Percutaneous trigeminal rhizotomy
SM: Spetzler-Martin
